# Examining environmental contaminant mixtures among adults with type 2 diabetes in the Cree First Nation communities of *Eeyou Istchee*, Canada

**DOI:** 10.1038/s41598-019-52200-x

**Published:** 2019-11-04

**Authors:** Aleksandra M. Zuk, Leonard J. S. Tsuji, Evert Nieboer, Ian D. Martin, Eric N. Liberda

**Affiliations:** 1 Health Studies, and the Department of Physical and Environmental Sciences, University of Toronto Scarborough, Toronto, Ontario Canada; 2 Department of Biochemistry and Biomedical Sciences, McMaster University, Hamilton, Ontario Canada; 3 School of Occupational and Public Health, Ryerson University, Toronto, Ontario Canada

**Keywords:** Type 2 diabetes, Environmental impact

## Abstract

Type 2 diabetes mellitus (T2DM) disproportionately affects Indigenous populations. It is possible that exposure to complex mixtures of environmental contaminants contribute to T2DM development. This study examined the association between complex environmental contaminant mixtures and T2DM among Canadian Indigenous communities from the *Eeyou Istchee* territory, Quebec, Canada. Using data from the cross-sectional Multi-Community Environment-and-Health Study (2005–2009) Principal Component Analysis (PCA) was used to reduce the dimensionality of the following contaminants: 9-polychlorinated biphenyl congeners; 7-organic pesticides; and 4-metal/metalloids. Following this data reduction technique, we estimated T2DM prevalence ratios (PR) and 95% confidence intervals using modified Poisson regression with robust error variance across derived principal components, adjusting for *a priori* covariates. For both First Nation adult males (n = 303) and females (n = 419), factor loadings showed dichlorodiphenyltrichloroethane (DDT) and lead (Pb) highly loaded on the second principal component (PC) axis: DDT negatively loaded, and Pb positively loaded. T2DM was significantly associated with PC-2 across all adjusted models. Because PCA produces orthogonal axes, increasing PC-2 scores in the fully adjusted model for females and males showed (PR = 0.84; 95% CI 0.72, 0.98) and (PR = 0.78; 95% CI 0.62, 0.98), respectively. This cross-sectional study suggests that our observed association with T2DM is the result of DDT, and less likely the result of Pb exposure. Further, detectable levels of DDT among individuals may possibly contribute to disease etiology.

## Introduction

Globally, diabetes continues to be a growing concern^1^. In Canada, Indigenous peoples are disproportionately affected by diabetes mellitus (T2DM)^2^. The lifetime risk of diabetes is estimated to be 8 in 10 among First Nations persons, and 5 in 10 among non-First Nations persons over 18 years of age^2^. However, the etiology and pathogenesis of diabetes mellitus is yet to be fully understood. Exposure to environmental contaminants and the risk of diabetes has received much research attention as persistent organochlorine pollutants (POPs) have been shown to be associated with type 2 diabetes mellitus^3–7^.

In an extensive review of predominantly cross-sectional studies, Taylor *et al*.^7^ reports a positive association between T2DM and some organochlorine pollutants (e.g., trans-nonachlor, dichlorodiphenyldichloroethylene (DDE), polychlorinated biphenyls (PCBs)). Similarly, in an updated, and globally-relevant review, Kuo *et al*.^8^ confirmed the positive association between organochlorine compounds (OCs) and T2DM. More specifically, Pal *et al*.^9^ observed higher plasma concentrations of OCs among persons with diabetes from First Nation communities in northern Canada. Similar to organic contaminants, long-term environmental exposure to toxic metals and/or deficiency of essential metals may possibly also contribute to the development of diabetes^10^. The role of various inorganic metals and metalloids on type 2 diabetes is complex. For example, Khan and Awan^10^ note that poor glycemic control and diabetes may alter the level of various essential trace elements due to polyuria. Chen *et al*.^11^ suggested that some heavy metals may play a role in T2DM etiology by adversely affecting islet function. Cross-sectional data show that metals such as cadmium may contribute to hypertriglyceridemia^12^. Commonly, type 2 diabetes is complicated with dyslipidemia and other factors associated with metabolic conditions which increase the risk of developing cardiovascular diseases and T2DM among Indigenous populations^13^. Further, toxic metals may act as endocrine disrupters that contribute to adiposity^14^, which is a risk factor that exacerbates metabolic and physiologic abnormalities associated with T2DM.

In Canada, Indigenous populations have a higher risk of developing T2DM and health-related complications compared to general Canadian population^2,15^. Therefore, examining the associations between environmental contaminants and T2DM is a priority, especially among Indigenous communities, where higher body burdens of complex mixtures exist. In this study, we examined the association between complex environmental contaminant mixtures and prevalent type 2 diabetes status among Canadian Cree communities residing in the *Eeyou Istchee* territory, in northern Quebec, Canada.

## Materials and Methods

### Data sources

**Figure 1:**
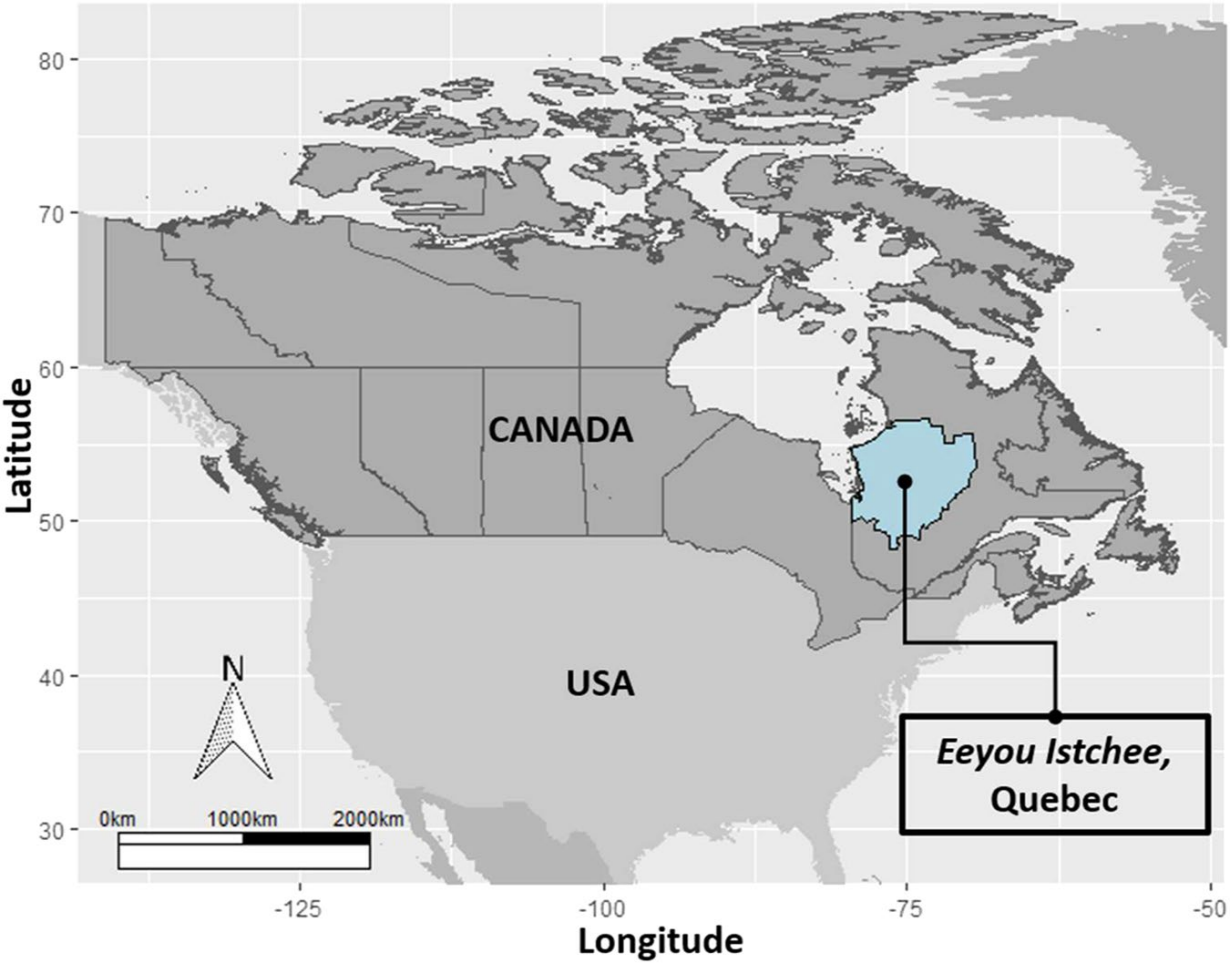
Eeyou Istchee Territory, Quebec, Canada.

The *Eeyou Istchee* territory, located in the James Bay Region of northern Quebec, Canada consists of nine Cree communities (Fig. 1). The *Nituuchischaayihtitaau Aschii* – Multi-Community Environment-and-Health Study aim was to provide assessment and surveillance among people of Eeyou Istchee. Eligibility for enrollment in the study included any person living on reserve. The Environment-and-Health Study stratified participants by age: children (0–7 years, and 8–14 years), adults (15–39 years, and 40 years and older). The main objectives examined the health effects of lifestyle factors, (including diet), environmental contaminants exposure, and environmental change on wildlife and aquatic ecosystems resulting from mining, forestry, and hydro-electric developments. In total, nine Cree communities were sampled. However, two of the nine communities were for an initial pilot study on preliminary health assessments conducted between 2002–2005, and not part of the analysis undertaken in this study. The remaining seven communities were studied between 2005–2009, which focused on participant health measures including exposure to environmental contaminates. Due to the time required to travel to between remote communities and collect all the necessary data, field data collection took place a two-to-four-week period over spring/summer. Specifically, one community was sampled in 2005, two in 2007, two in 2008, and two in 2009 (community names withheld at the request of the Cree Board of Health and Social Services of James Bay. Full details about the Multi-Community Environment-and-Health Study are provided^16–19^. As part of the *Nituuchischaayihtitaau Aschii* – Multi-Community Environment-and-Health Study, trained research nurses were integral to the study data collection. Participants underwent a physical examination, completed health and dietary surveys, and provided tissue and blood samples for laboratory analysis. An additional medical chart review was performed by a research nurse who had been involved in the clinical field work to verify individual health-related information ascertained from health-questionnaires for all consenting adults. Informed consent was obtained from all participants or their guardians in Cree, English, or French. The *Nituuchischaayihtitaau Aschii* – Multi-Community Environment-and-Health Study was conducted in accordance with relevant guidelines, regulations, and research agreements. All work conducted was approved by the research ethics boards of McGill University and Laval University, in partnership with the Cree Board of Health and Social Services of James Bay and McMaster University.

### Study population

In the 2005–2009 Environment-and-Health Study, 1750 participants were recruited. Our analysis included the following adults over the age of 20 years of age who had: (1) medical-chart verified T2DM diagnoses; (2) complete environmental contaminant exposure profiles; (3) undergone physical examination, completed interviewed health questionnaires, and underwent a phlebotomy blood draw were retained for analyses. Medical chart reviews were conducted in only seven of the nine communities for self-reported medical conditions. Therefore, adults who had not undergone medical chart review were excluded from analysis. Adults with type 1 diabetes were also excluded. This resulted in a total of 722 cases, representing seven of the nine communities from the *Eeyou Istchee* territory. A flow chart of the sample is presented in the Supplemental Fig. S1.

### Environmental contaminant analyses

Details concerning the analytical methods and related QA/QC are provided in Liberda *et al*.^20^. Briefly, OCs were recovered from blood plasma using solid-phase extraction and cleaned on a florisil columns prior to high resolution gas chromatography-mass spectrometry (HRGC-MS) analysis. Limits of detection (LODs) were based on a signal-to-noise ratio of 3:1 also as previously reported. Polychlorinated biphenyl (PCBs) congeners (CBs 99, 187, 183, 180, 170, 153, 128, 118, and 105), organic pesticides (cis-Nonachlor, Dichlorodiphenyltrichloroethane [*p,p’-*DDT], Dichlorodiphenyldichloroethylene [*p,p’-*DDE], Hexachlorobenzene [HCB], Mirex, oxy-chlordane, trans-Nonachlor) were all assessed for their concentrations.

Whole blood samples were drawn from participants to measure concentrations of a selection of elements (Lead [Pb], total mercury [Hg], cadmium [Cd], and selenium [Se]) and were kept frozen until analyzed at the Institut National de Santé Publique du Québec (INSPQ) Human Toxicology Laboratory using inductively coupled plasma mass spectrometry (ICP–MS) as detailed in Nieboer *et al*.^21^. All limits of detection (LODs) and the analytical methods are also described therein.

Contaminants that were detected in less than 10% of the total participants were excluded from analyses. Several methods exist for imputing missing values, however, the most common methods used for imputing samples with limits below the detection limit are using half the detection limit or one over the square root of two multiplied by the detection limit^22^. Non-detections for all individuals’ contaminant body burdens were imputed as half the detection limit as is recommended by the United States Environmental Protection Agency^23^. Due to year-to-year analytical detection limit variation (i.e., lower detection limits due to better technology and standards), we utilized the highest detection limit through all years to prevent false differences owing the improvement of the limit of detection overtime, and hence community.

### Outcome assessment

Health information was initially obtained through interviewer-administered questionnaires. Medical chart reviews were conducted for each consenting participant to confirm and gain additional health information (i.e., medication use and medical history). All participants who were diagnosed with type 2 diabetes were confirmed through medical chart review.

### Risk factor covariates

Covariate measures were ascertained through either self-report, by interviewer administered health questionnaires, or via direct physical examination, which included a blood draw. Detailed aspects of each are provided in Nieboer *et al*.^19^. Age was categorized into the following groups: 20–39, 40–59, and ≥60 years. Educational attainment was self-reported and defined according to the following groups: completed less than high school, high school, and some or more college. The survey questionnaire collected information on smoking habits, which classified participants as “current, former and never smoker.” Due to the low prevalence of ‘never-smokers” in our analysis smoking status is a composite measure of “current and occasional smokers” compared to “former or non-smokers. Standing height and body weight was measured at the time of the physical examination. Body mass index (BMI) was calculated according to weight in kilograms (kg) divided by height measures (meters squared, m^2^). Total lipids concentrations were determined using methods described by Rylander *et al*.^24^.

### Statistical methods

#### Statistical analysis

Descriptive statistics were calculated for all contaminant concentrations and covariates, stratified by sex and diagnosis of T2DM. Continuous variables were reported as means ± standard deviations (SD) or geometric means, where appropriate. Categorical data are reported as frequencies and percentages. Using SAS PROC GENMOD procedures, we separately estimated adjusted prevalence ratios (PR) using modified Poisson regression with robust error variance^25,26^. Multivariable models examined the association between T2DM (a non-rare binary outcome) and derived principal components (PCs) adjusted for the following *a priori* covariates: age, plasma lipid concentrations, BMI, smoking status, and education. Overall, the following covariates were missing among females and males, respectively; education: 1.7% (n = 7) and 2.97% (n = 9); BMI: 1.9% (n = 8) and 5.3% (n = 16) and; smoking status: 1.4% (n = 6) and 2.97% (n = 9). Consequently, numbers of individuals in subsequent regression analyses were reduced slightly depending upon the number of valid observed covariates. Statistical analyses were carried out using SAS v9.4 (SAS Institute, Inc., Cary, NC) and all figures were generated using R (version 3.5.2; Vienna, Austria).

#### Principal component analysis

Principal component analysis (PCA) was used to transform an initial set of 21 plasma or whole blood contaminant variables (i.e., PCB congeners, organic pesticides, and metals/metalloid) into a reduced number of uncorrelated (i.e., orthogonal) predictor variables by maximizing the variance of the original variables into derived fewer dimensions or principal components (PC)^27,28^. We used the correlation matrix of contaminant variables as the input matrix for PCA, and put all original variables on a common scale. Components with eigenvalues exceeding 1.0 were retained and used to define independent summary axes. Therefore, the first principal component axes (i.e., PC-1) will account for the largest variance in the data, and any subsequent PCs (i.e., orthogonal to the first) will account for a portion of the variance not accounted for in the preceding component. The new derived PCs (i.e., scores) are linear combinations of all original variables. Values, or scores, for individuals on these new PC variables are measures of shared exposure to the original contaminant concentrations. Prior to the PCA, contaminant concentrations were log10-transformed (variate + 1), improving normality of the distribution^29,30^. Separate PCAs were performed for female and male cohorts, owing to differential prevalence of T2DM between sexes and differing levels of exposure for females and males^31^. Absolute component loadings of 0.50 or greater were identified as important for a given principal component. Thus, signs of loadings are arbitrary, only the relative magnitude and patterns are meaningful^32^. Separate-sex principal component (PC) scores summarized new, synthetic measures of contaminant burdens for both males and females. These uncorrelated PC score variables were then used as independent predictors in the regression analysis of T2DM.

#### Sensitivity analysis

Based on findings from the regression models, we examined the frequency of detection of two variables (i.e., DDT and Pb) by diagnosed T2DM status in contingency analysis. Adjusted Standardized Residuals (ARS) of contaminant levels measured above or below the limit of detection were calculated for T2DM. An association between the frequency of detectability of contaminants, above or below the limit of detection with T2DM status was explored by examining overall chi-square significance and ASRs greater than (|1.96|) in a 2 × 2 contingency table. Complete-case analysis was also performed as a sensitivity check, which found no appreciable difference in results using the same modified Poisson regression models^33^.

### Ethics approval and consent to participate

All work conducted was approved by the research ethics boards of McGill University and Laval University, in partnership with the Cree Board of Health and Social Services of James Bay and McMaster University. Informed consent was obtained from all participants or their guardians in Cree, English, or French.

## Results

### Descriptive results

Summary statistics of Cree population data for demographic, risk factors variables and contaminants are presented in Table 1. In total, there were 722 participants, 419 females, and 303 males. The prevalence of T2DM among females and males was 23% (n = 95) and 16.5% (n = 50), respectively.

**Table 1:** Participant characteristics according to sex and type 2 diabetes status: results among *Nituuchischaayihtitaau Aschii* – Multi-Community Environment-and-Health Study (2005–2009).

Characteristics	Sex stratified	Total population (n = 722)	% ≤ LOD^a^	Type 2 Diabetes Status	
Demographic				Present	Absent
				N (%); or Mean ± SD	N (%); or Mean ± SD
Sex (n, %)	Female	419 (58%)		95 (23%)	324 (77%)
	Male	303 (42%)		50 (16.5%)	253 (83.5%)
Age (years)	Female			47.9 ± 14.7	38.5 (13.9)
	Male			56.2 ± 14.9	40.6 ± 14.6
**Education**					
Less than High school	Female	412		34 (37.4%)	55 (17.1%)
Some or completed High school				35 (38.5%)	181 (56.4%)
Some or completed College or higher (R)				22 (24.2%)	85 (26.5%)
Less than High school	Male	294		19 (40.4%)	47 (19.0%)
High school				20 (42.6%)	156 (63.2%)
Some College or higher				8 (17.0%)	44 (17.8%)
**Risk Factors**					
**Anthropometry**					
BMI (kg/m^2^)	Female	411		39 ± 8.6	34.5 ± 6.5
	Male	287		34.5 ± 5.8	31.7 ± 5.6
**Health factors**					
Smoking status, current/occasional smoker compared to former/never (R)	Female	413		28 (30.8%)	176 (54.7%)
	Male	294		6 (12.8%)	127 (51.4)
**Cardiometabolic**					
Total lipids (g/L)^b^	Female	419		6.4 ± 1.6	5.8 ± 1.1
	Male	302		5.9 ± 1.1	6.5 ± 1.9
**Contaminants (µg/L)** ^**c**^					
PCB 99	Female		40.6%	0.116 ± 4.061	0.044 ± 3.470
	Male		32.3%	0.108 ± 3.333	0.054 ± 3.438
PCB 105	Female		49.2%	0.058 ± 3.331	0.025 ± 2.563
	Male		47.2%	0.046 ± 2.832	3.438 ± 2.345
PCB 118	Female		15.7%	0.262 ± 4.583	0.074 ± 4.508
	Male		10.9%	0.236 ± 3.930	0.086 ± 3.991
PCB 128	Female		83.3%	0.019 ± 1.639	0.015 ± 1.331
	Male		77.6%	0.017 ± 1.490	0.016 ± 1.403
PCB 138	Female		5.0%	0.565 ± 4.641	0.175 ± 5.077
	Male		1.6%	0.678 ± 3.854	0.275 ± 4.404
PCB 153	Female		1.4%	1.204 ± 4.877	0.376 ± 5.586
	Male		0.3%	1.695 ± 4.185	0.666 ± 4.686
PCB 170	Female		16.9%	0.259 ± 4.592	0.093 ± 4.751
	Male		6.6%	0.407 ± 4.264	0.168 ± 4.485
PCB 180	Female		2.63%	0.887 ± 5.069	0.290 ± 5.677
	Male		1.3%	1.483 ± 4.583	0.572 ± 4.998
PCB 183	Female		30.1%	0.112 ± 3.919	0.044 ± 3.442
	Male		17.8%	0.131 ± 3.570	0.063 ± 3.520
PCB 187	Female		13.4%	0.348 ± 4.863	0.117 ± 5.077
	Male		6.3%	0.528 ± 4.293	0.212 ± 4.695
cis-Nonachlor	Female		50.6%	0.052 ± 2.945	0.024 ± 2.405
	Male		38.0%	0.064 ± 3.074	0.031 ± 2.628
p,p’-DDE	Female		0.7%	2.958 ± 3.305	1.043 ± 3.746
	Male		0.3%	2.993 ± 2.911	1.386 ± 2.866
p,p’-DDT	Female		88.3%	0.035 ± 1.811	0.027 ± 1.326
	Male		90.0%	0.031 ± 1.626	0.027 ± 1.372
Hexachlorobenzene (HCB)	Female		31.5%	0.120 ± 3.054	0.055 ± 2.878
	Male		21.8%	0.120 ± 2.476	0.071 ± 2.666
Mirex	Female		25.1%	0.161 ± 4.620	0.062 ± 4.432
	Male		14.2%	0.266 ± 4.681	0.113 ± 4.934
oxy-Chlordane	Female		30.8%	0.085 ± 3.249	0.035 ± 2.862
	Male		18.5%	0.104 ± 3.103	0.048 ± 2.851
trans-Nonachlor	Female		20.8%	0.150 ± 3.527	0.052 ± 3.510
	Male		9.2%	0.212 ± 3.456	0.084 ± 3.422
Cadmium, Cd (nmol /L)	Female		40.1%	5.563 ± 2.729	8.849 ± 2.782
	Male		51.2%	3.926 ± 2.338	8.192 ± 3.104
Total mercury, Hg (nmol /L)	Female		15.7%	28.275 ± 3.798	14.862 ± 3.809
	Male		13.2%	44.208 ± 3.383	20.433 ± 3.990
Lead, Pb (µmol /L)	Female		29.1%	0.131 ± 2.848	0.119 ± 3.003
	Male		9.2%	0.164 ± 2.680	0.196 ± 2.528
Selenium, Se (µmol /L)	Female		0.2%	2.209 ± 1.218	2.118 ± 1.157
	Male		0.3%	2.244 ± 1.151	2.235 ± 1.140

Missing values among adult females; Education (n = 7, 1.7%); BMI (n = 8, 1.9%); Smoking status (n = 6, 1.4%). Missing values among adult males; Education (n = 9, 2.97%); BMI (n = 16, 5.3%); Smoking status (n = 9, 2.97%).

**Abbreviations: N**, frequency value; **%**, percentage; **BMI**, Body mass index; **R**, reference category; **PCB**, Polychlorinated biphenyl congeners; ***p,p’-*DDT**, Dichlorodiphenyltrichloroethane; ***p,p’-*DDT**, Dichlorodiphenyldichloroethylene;

^a^Percentage of contaminants below the level of detection (LOD).

^b^Total lipid concentrations were determined using methods described by Rylander *et al*. 2012.

^c^Presented are geometric mean ± standard deviation (SD).

The mean age among participants with T2DM was 47.9 years and 56.2 years for females and males with diagnosed type 2 diabetes, respectively. Among female respondents, 24.2% self-reported attaining some or more college education whereas among males, 17% had attained some form of college education. Among adults with diabetes, body mass index (BMI) at the time of examination was higher for females (39 kg/m^2^) than males (34.5 kg/m^2^). As well, there was a two-fold higher prevalence of self-reported smoking status (i.e., current and occasional compared to former or never) among females with T2DM. The total mean lipid concentrations also differed among females and males among adults with diagnosed T2DM, 6.4 g/L and 5.9 g/L, respectively.

### Contaminant principal component analysis (PCA) loadings

**Figure 2:**
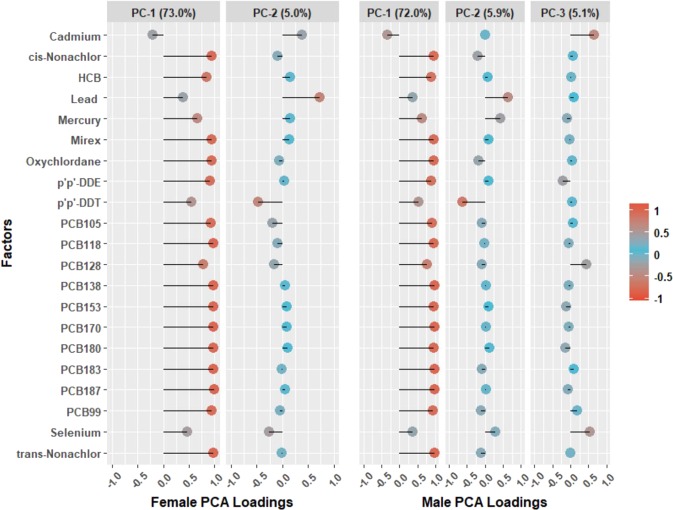
Principal Component (PC) Loadings for Males and Females

Sex-stratified contaminant PC loadings are shown in Fig. 2. Among females, eigenvalues greater than 1 were found for the first two components. PC-1 explained 73% of the total variance in the original log transformed concentrations, which for increasing PCA scores, resulted in high positive loadings for PCBs, organochlorines, and Hg. On the second axis, PC-2 accounted for 5% of the variation, showing that DDT had a negative loading relative to the positive loading of Pb on PC-2 among females. However, for decreasing PCA scores, DDT loadings are interpreted as positive relative to Pb, which is interpreted as having negative loadings.

Among males, contaminant PCA revealed three orthogonal axes, which explained 72%, 6%, and 5%, of the variation for PC-1, PC-2, and PC-3, respectively. Similar to females, PC-1 was highly positively loaded by PCBs, most organochlorines, and Hg. DDT had a strong negative loading, but high positive loading for lead Pb, and a moderate loading for Hg for the second PC axis. Lastly, cadmium and selenium loaded positively on the third PC axis for males.

**Figure 3:**
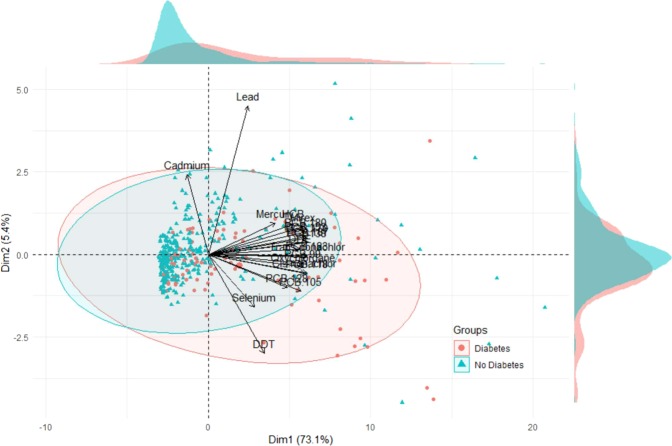
PCA Biplot of Principal Component (PC) Axes and Type 2 Diabetes Status among both Males and Females.

PCA biplot of orthogonal PC axes overlaid with T2DM status among Indigenous Cree adults in the *Eeyou Istchee* territory (Fig. 3).

### Main effects of the association between PCA scores and type 2 diabetes

Multivariable modified Poisson regression analyses are presented in Tables 2 and 3, which investigates the relationship between prevalent T2DM and the extracted orthogonal principal components of contaminant exposure, for both adult females and males, respectively. Among adult females, the prevalence ratio for PC-2 (but not for PC-1), was significantly associated with T2DM across all adjusted models. After fully adjusting for covariates, the final model for females shows, PC-2 (PR = 0.84; 95% CI: 0.72, 0.97) was significantly associated with T2DM. For increasing PC-2 scores, DDT negatively loaded, and Pb was positively loaded, on the second axes. Therefore, as PC-2 axis scores increase (i.e., DDT loadings decreases and Pb loadings increases), resulting in a PR significantly less than 1. Conversely, as PC-2 axis score decreases (i.e., DDT loadings increase and Pb loadings decrease), PC-2 is significantly associated with prevalent T2DM (PR = 1.19; 1.03, 1.38) among females. Similarly, among adult males, PC-2 (explaining 5% of variation) also was significantly associated with prevalent T2DM across all adjusted models. PC-2 was shown to have a strong positive and negative loading for Pb and DDT, respectively. In the fully adjusted model, PC-2 was significantly associated with T2DM (RR = 0.78, 95%; CI: 0.62–0.98). As above, since a decrease in PC-2 score indicates an increase in DDT loadings, resulting in a similar significant association between (PR = 1.27; 95% CI: 1.02–1.60).

**Table 2:** Multivariable adjusted prevalence ratios (95% Confidence Intervals) for type 2 diabetes mellitus and orthogonal principal component axes among adult females over 20 years of age using data from the *Nituuchischaayihtitaau Aschii* – Multi-Community Environment-and-Health Study (2005–2009)

Exposure variables	Total frequency (N)	Prevalence Ratio (PR)	95% CI	95% CI	P value*
**Model 1**	419		**Lower limit**	**Higher limit**	
PC1		1.13	0.92	1.39	0.2542
PC2		0.79	0.69	0.91	0.0008*
**Model 2**	419				
PC1		1.08	0.87	1.34	0.4716
PC2		0.80	0.70	0.91	0.0011*
**Model 3**	411				
PC1		1.07	0.85	1.35	0.5592
PC2		0.83	0.72	0.95	0.0090*
**Model 4**	410				
PC1		1.12	0.90	1.40	0.3047
PC2		0.85	0.73	0.98	0.0285*
**Model 5**	409				
PC1		1.07	0.84	1.36	0.6009
PC2		0.84	0.72	0.98	0.0220*

Abbreviations: CI, confidence intervals; PC1 and PC2, first and second orthogonal principal component axes, respectively.

*Significance (p < 0.05).

Model 1: adjusted for age;

Model 2: Model 1, plus lipids

Model 3: Model 2, plus body mass index.

Model 4: Model 3, plus smoking status.

Model 5: Model 4, plus education.

**Table 3:** Multivariable adjusted prevalence ratios (95% Confidence Intervals) for type 2 diabetes mellitus and orthogonal principal component axes among adult males over 20 years of age using data from the *Nituuchischaayihtitaau Aschii* – Multi-Community Environment-and-Health Study (2005–2009).

Exposure variables	Total frequency (N)	Prevalence Ratio (PR)	95% CI	95% CI	p value*
**Model 1**	303		**Lower limit**	**Higher limit**	
PC1		0.90	0.66	1.22	0.4805
PC2		0.82	0.69	0.97	0.0220*
PC3		0.78	0.59	1.02	0.0659
**Model 2**	303				
PC1		0.94	0.68	1.32	0.7376
PC2		0.76	0.64	0.91	0.0033*
PC3		0.83	0.62	1.10	0.1866
**Model 3**	286				
PC1		0.97	0.69	1.37	0.8625
PC2		0.79	0.64	0.98	0.0291*
PC3		0.86	0.63	1.19	0.3783
**Model 4**	284				
PC1		0.94	0.69	1.27	0.6785
PC2		0.78	0.62	0.97	0.0260*
PC3		1.02	0.73	1.42	0.9225
**Model 5**	282				
PC1		0.96	0.68	1.37	0.8271
PC2		0.78	0.62	0.98	0.0363*
PC3		1.01	0.72	1.40	0.9710

Abbreviations: CI, confidence intervals; PC1, PC2, and PC3, first, second and third orthogonal principal component axes, respectively.

*Significance (p < 0.05).

Model 1: adjusted for age;

Model 2: Model 1, plus lipids.

Model 3: Model 2, plus body mass index.

Model 4: Model 3, plus smoking status.

Model 5: Model 4, plus education.

### Sensitivity analysis results

Based on the significant associations observed in PC-2, we further explored the role of DDT and Pb on T2DM. Rather than examining only concentration values, we inspected the frequency of detection for DDT and Pb on T2DM status by contingency analysis. An examination of the adjusted standardized residuals showed that adults with T2DM were significantly more often than expected to have detectable levels of DDT (females: 55%, ASR 5.8, p < 0.001; males: 30%, ASR 2.1, chi-square p = 0.036) compared to only 18.4% (ASR −5.8) and 15% (ASR −2.1) with levels below detection for DDT among females and males, respectively. Lead (Pb) detection frequencies between T2DM states were not found to be statistically significant. Single-pollutant models for DDT and Pb levels are also provided in the Supplemental Material to aid interpreting of the PCA results (Supplementary Material, Table S4).

## Discussion

In this cross-sectional Multi-Community Environment-and-Health Study among adult Indigenous peoples, we show that DDT and Pb load oppositely to each other on the second component axis indicating differential exposures. PCA has distributed the variation of the contaminants on what appears to be similar groups of lipophilic or hydrophilic compounds and/or their sources. As DDT and Pb are considered together on the second axis, decreasing PC-2 axes scores manifest as increasing DDT and decreasing Pb loadings. Positive DDT loadings were associated with type 2 diabetes with decreasing axes scores for both females and males.

Experimentally, it has been shown that perinatal DDT exposure in mice may contribute to insulin resistance and metabolic syndrome in adult female offspring^34^. A separate experiment in male mice exposed to DDT reported significant reductions in glucose tolerance and pancreatic activity^35^. More recently, a systematic review and meta-analysis of observational human studies reported a significant overall increased risk between DDT and type 2 diabetes (odds ratio 1.79 [95% CI 1.31, 2.4]^36^. Additionally, exposure to other organochlorine pesticides were also shown to be associated with T2DM^36^. However, specific sex-related differences were not examined in the systematic review, and the responsible mechanisms of action for sex-dependent findings remains yet to be elucidated. One possible mechanism may be due to DDT’s role as an estrogen agonist and an androgen antagonist^34,37^.

This is the first study to examine complex body burdens using a reduction method technique on prevalent type 2 diabetes among Indigenous peoples in the *Eeyou Istchee* territory of northern, Quebec. The combined biological adverse effects of body burden are a concern to human health, particularly among areas of northern Canada, where environmental contaminants are reported to be present, often at greater concentrations^38–40^. Glucose dysregulation has been shown to be associated with organochlorine compounds, though not consistently in the literature. In a meta-analysis of 23 studies conducted globally (i.e., 18 cross-sectional and 4 prospective), Tang *et al*.^41^ show that organochlorine pollutants (OCPs) were significantly associated with type 2 diabetes. Pooled estimates across the studies revealed substantial heterogeneity, and DDT’s primary metabolite, *p,p’*-DDE modestly increased the odds of diabetes^41^. Comparably, Magliano *et al*.^42^ epidemiological review of persistent organic pollutants also report an independent association with diabetes. For example, using nationally representative data from the U.S. the National Health and Nutrition Examination Survey (NHANES) report that after adjusting for covariates, detectable concentration exposure categories of DDE (e.g., 75^th^ and 90^th^ percentiles) when compared to the lowest reference group had 2- and 4-fold significant association with prevalent diabetes, respectively^43^. Similarly, using NHANES data, Everett *et al*.^44^ found that high levels of serum DDT levels were significantly associated with both undiagnosed, and total diabetes (diagnosed plus undiagnosed). Moreover, among high-risk populations in areas known to be heavily polluted, Indigenous (Mohawk) Americans, and First Nations of northern Ontario had similar results between *p,p’*-DDE and diabetes, although following covariate adjustment, lost statistical significance^45,46^. Overall, our results support previous studies showing evidence of an association between DDT/*p,p’*-DDE and diabetes risk^47^. Additionally, our study shows that both males and females diagnosed with T2DM had significantly higher frequency of detection of DDT that was not observed for Pb. Subsequently, contaminant concentration may not be the sole contributing factor, but possibly rather more exposure status (i.e., detects versus non-detections) may potentially play a role in disease etiology. Additionally, not all studies have found an association between persistent organic pollutants and type 2 diabetes risk. For example, in a comparable cross-sectional study among Inuit population in Greenland found no association between POPs and prevalent diabetes, or impaired glucose tolerance^48^. In a small exploratory study among First Nations adults found that some PCBs (153, 74) from fish consumption were associated with self-reported type 2 diabetes^46^. In our analysis using a similar population we did not find an association between PCBs and type 2 diabetes. Furthermore, in a study that examined incidence of diabetes in a cohort of Great Lakes sport fish consumers, years of eating sport fish was not found to be associated with incident diabetes, but DDE exposure (breakdown product of DDT) was associated with incident diabetes^49^, which was not found in our cross-sectional study. Interestingly, in a case-control study among older women from the Sweden, PCB 153, and *p,p*′-DDE was not initially associated with type 2 diabetes. However, when a small sample of these older adults was analyzed with diabetes diagnosed more than six years from baseline, the highest quartile of CB-153 and *p,p*′-DDE showed an increased risk of type 2 diabetes^50^.

Blood lead levels strongly contributed to the second principal component axes, which reflects lead-containing ammunition exposure used for hunting traditional foods^39,40^. Experimentally, blood Pb has been shown to alter glucose metabolism in obese murine models^51^. Lead may possibly contribute to diabetes since Pb is a pro-oxidant, and oxidative stress alters and reduces insulin signaling^51^. In humans, few studies have explored the contribution of blood Pb levels on diabetes risk^51,52^. However, exposure studies report that Pb levels are higher in persons with diabetes, though findings are inconsistent^52,53^. Hectors *et al*.^54^ note that it is possible that individuals with diabetes may have excretory or metabolic deficiencies resulting in higher body burdens of environmental contaminants, or that exposure compounds themselves contribute to the development of diabetes. Additionally, Pb has been shown to disrupt metabolic function in the co-occurrence of diabetes-related comorbidities such as non-alcoholic fatty liver disease and impaired renal function^52,55,56^.

Our study has several strengths. First, by utilizing PCA as a data reduction tool, we were able to isolate the unique exposure pattern of one of the organic contaminants in a complex mixture that may be playing a significant role in the etiology and morbidity of T2DM. Second, we explored frequency of detections of DDT, which may potentially have on disease etiology. However, there are several key limitations. First, it is important to note that these associations could have been observed due to specific unmeasured lifestyle aspects, which cause a co-exposure to DDT. Second, we cannot infer temporality between contaminant exposures on risk of T2DM, as the data are cross-sectional. Thus, more research into how DDT may play a role in T2DM is needed. Third, residual confounding by smoking is a potential concern given that it was necessary to create a composite smoking status measure of two broad categories due to the low prevalence of non-smokers. Lastly, because PCA is a linear combination of all original variables, which project orthogonal axes through the data, this may complicate the interpretation of the results. We have therefore provided additional details in the results section, as well as highlighted the key single-pollutant models in the Supplementary Material.

In conclusion, owing to the complex nature of all contaminants loadings on orthogonal PCA, we posit that our observed association with type 2 diabetes is consistent with increased exposure to DDT over the influence of exposure to other organic or metal contaminants, and less likely the result of Pb exposure among Indigenous Cree adults in the *Eeyou Istchee* territory.

## Supplementary information

Supplementary information

## Data Availability

Datasets generated and analyzed for this study are available through the Cree Board of Health and Social Services of James Bay.

## References

[1] World Health Organization. Diabetes. Available at: https://www.who.int/news-room/fact-sheets/detail/diabetes (Accessed: 12th March 2019) (2018).

[2] Crowshoe L (2018). Type 2 Diabetes and Indigenous Peoples. Can. J. Diabetes.

[3] Ngwa EN, Kengne A-P, Tiedeu-Atogho B, Mofo-Mato E-P, Sobngwi E (2015). Persistent organic pollutants as risk factors for type 2 diabetes. Diabetol. Metab. Syndr..

[4] Lee Y-M, Jacobs DR, Lee D-H (2018). Persistent Organic Pollutants and Type 2 Diabetes: A Critical Review of Review Articles. Front. Endocrinol. (Lausanne)..

[5] Zong G (2018). Persistent organic pollutants and risk of type 2 diabetes: A prospective investigation among middle-aged women in Nurses’ Health Study II. Environ. Int..

[6] Henríquez-Hernández LA (2017). Persistent organic pollutants and risk of diabetes and obesity on healthy adults: Results from a cross-sectional study in Spain. Sci. Total Environ..

[7] Taylor KW (2013). Evaluation of the Association between Persistent Organic Pollutants (POPs) and Diabetes in Epidemiological Studies: A National Toxicology Program Workshop Review. Environ. Health Perspect..

[8] Kuo C-C, Moon K, Thayer KA, Navas-Acien A (2013). Environmental Chemicals and Type 2 Diabetes: An Updated Systematic Review of the Epidemiologic Evidence. Curr. Diab. Rep..

[9] Pal S (2013). The association of type 2 diabetes and insulin resistance/secretion with persistent organic pollutants in two First Nations communities in northern Ontario. Diabetes Metab..

[10] Khan A, Awan F (2014). Metals in the pathogenesis of type 2 diabetes. J. Diabetes Metab. Disord..

[11] Chen YW (2009). Heavy metals, islet function and diabetes development. Islets.

[12] Chateau-Degat M-L (2009). Diabetes and related metabolic conditions in an aboriginal cree community of quebec, Canada. Can. J. diabetes.

[13] Rask-Madsen C, Kahn CR (2012). Tissue–Specific Insulin Signaling, Metabolic Syndrome, and Cardiovascular Disease. Arterioscler. Thromb. Vasc. Biol..

[14] Davis RAH, Plaisance EP, Allison DB (2018). Complementary Hypotheses on Contributors to the Obesity Epidemic. Obesity (Silver Spring)..

[15] Rice K (2016). Best Practices for the Prevention and Management of Diabetes and Obesity-Related Chronic Disease among Indigenous Peoples in Canada: A Review. Can. J. Diabetes.

[16] Pereg, D. & Nieboer, E. Nituuchischaayihtitaau Aschii Multi-community Environment-and-Health Longitudinal Study in Iiyiyiu Aschii: Mistissini. Technical report: summary of activities, results and recommendation. Available at: http://www.creehealth.org/library/online/research/environmental-health-study-technical-report-mistissini. (2007).

[17] Nieboer, E. & Robinson, E. Nituuchischaayihtitaau Aschii Multi -community environment -and -health longitudinal study in Eeyou Istchee: Eastmain and Wemindji., 978-2-550-61860-7 (2007).

[18] Nieboer, E., Pereg, D., Bonnier-Viger, Y., Eageland, G. & Dewailly, E. *Nituuchischaayihtitaau Aschii Multi-community Environment-and-Health Study in Iiyiyiu Aschii: Mistissini*. (2007).

[19] Nieboer, E. *et al*. Nituuchischaayihtitaau Aschii Multi-community Environment-and-Health Study in Eeyou Istchee 2005–2009: Final Technical Report. *Public Health Report Series 4 on the Health of the Population. Chisasibi QC: Cree Board of Health and Social Services of James Ba* 1–191 Available at: http://www.creehealth.org/sites/default/files/E-and-HTechnicalReport.pdf (Accessed: 2nd September 2019) (2013).

[20] Liberda Eric N., Tsuji Leonard J.S., Martin Ian D., Cote Suzanne, Ayotte Pierre, Dewailly Eric, Nieboer Evert (2014). Plasma concentrations of persistent organic pollutants in the Cree of northern Quebec, Canada: Results from the multi-community environment-and-health study. Science of The Total Environment.

[21] Nieboer E (2017). Body burdens, sources and interrelations of selected toxic and essential elements among the nine Cree First Nations of Eeyou Istchee, James Bay region of northern Quebec, Canada. Environ. Sci. Process. Impacts.

[22] Helsel D (2010). Much ado about next to nothing: Incorporating nondetects in science. Annals of Occupational Hygiene.

[23] U.S. Environmental Protection Agency. Assigning values to non-detected/non-quantified pesticide residues in human health food exposure assessments. Available at: https://archive.epa.gov/pesticides/trac/web/pdf/trac3b012.pdf. Accessed 12 November 2018. 1–25 Available at: https://archive.epa.gov/pesticides/trac/web/pdf/trac3b012.pdf. (Accessed: 2nd September 2019) (2000).

[24] Rylander L (2012). Very high correlations between fresh weight and lipid-adjusted PCB-153 serum concentrations: Irrespective of fasting status, age, body mass index, gender, or exposure distributions. Chemosphere.

[25] Fang, J. Using SAS® Procedures FREQ, GENMOD, LOGISTIC, and PHREG to Estimate Adjusted Relative Risks – A Case Study. *SAS Glob. Forum 2011 Stat. Data Anal*. 345 (2011).

[26] Zou G (2004). A Modified Poisson Regression Approach to Prospective Studies with Binary Data. Am. J. Epidemiol..

[27] Thioulouse Jean, Dray Stéphane, Dufour Anne-Béatrice, Siberchicot Aurélie, Jombart Thibaut, Pavoine Sandrine (2018). Multivariate Analysis of Ecological Data with ade4.

[28] Legendre, P., Legendre, L., Legendre, L. & Legendre, P. *Numerical ecology*. (Elsevier, 2012).

[29] Huntley B., Gauch H. G., Gillison A. N., Anderson D. J. (1983). Multivariate Analysis in Community Ecology. The Journal of Ecology.

[30] Green, R. H. *Sampling design and statistical methods for environmental biologists*. (Wiley, 1979).

[31] Kuzmina, E., Lejeune, P., Dannenbaum, D. & Torrie, J. E. *Public Health Report Series 3 on Diabetes Cree Board of Health and Social Services of James Bay*. (2010).

[32] Jollife IT, Cadima J (2016). Principal component analysis: A review and recent developments. Philosophical Transactions of the Royal Society A: Mathematical, Physical and Engineering Sciences.

[33] Greenland S, Finkle WD (1995). A critical look at methods for handling missing covariates in epidemiologic regression analyses. American Journal of Epidemiology.

[34] La Merrill M (2014). Perinatal exposure of mice to the pesticide DDT impairs energy expenditure and metabolism in adult female offspring. PLoS One.

[35] Yau ET, Mennear JH (1977). The inhibitoty effect of DDT on insulin secretion in mice. Toxicol. Appl. Pharmacol..

[36] Evangelou E (2016). Exposure to pesticides and diabetes: A systematic review and meta-analysis. Environ. Int..

[37] Costa, L. Toxic Effect of Pesticides. In: *Casarett and Doull’s Toxicology: Basic Science of Poisons - 7th edition*., 10.1036/0071470514 (2008).

[38] Wainman, B. C. *et al*. Menstrual cycle perturbation by organohalogens and elements in the Cree of James Bay, Canada. *Chemosphere*, 10.1016/j.chemosphere.2015.12.056(2016). 10.1016/j.chemosphere.2015.12.056 PMC4827016 26855224

[39] Juric AK (2018). Risk assessment of dietary lead exposure among First Nations people living on-reserve in Ontario, Canada using a total diet study and a probabilistic approach. J. Hazard. Mater..

[40] Liberda ENEN (2018). Source identification of human exposure to lead in nine Cree Nations from Quebec, Canada (Eeyou Istchee territory). Environ. Res..

[41] Tang M, Chen K, Yang F, Liu W (2014). Exposure to Organochlorine Pollutants and Type 2 Diabetes: A Systematic Review and Meta-Analysis. PLoS One.

[42] Magliano DJ, Loh VHY, Harding JL, Botton J, Shaw JE (2014). Persistent organic pollutants and diabetes: A review of the epidemiological evidence. Diabetes Metab..

[43] Lee D-H (2006). A Strong Dose-Response Relation Between Serum Concentrations of Persistent Organic Pollutants and Diabetes: Results from the National Health and Examination Survey 1999–2002. Diabetes Care.

[44] Everett CJ (2007). Association of a polychlorinated dibenzo-p-dioxin, a polychlorinated biphenyl, and DDT with diabetes in the 1999–2002 National Health and Nutrition Examination Survey. Environ. Res..

[45] Codru N (2007). Diabetes in relation to serum levels of polychlorinated biphenyls and chlorinated pesticides in adult Native Americans. Environ. Health Perspect..

[46] Philibert A, Schwartz H, Mergler D (2009). An exploratory study of diabetes in a first nation community with respect to serum concentrations of p,p’-DDE and PCBs and fish consumption. Int. J. Environ. Res. Public Health.

[47] Lind PM, Lind L (2018). Endocrine-disrupting chemicals and risk of diabetes: an evidence-based review. Diabetologia.

[48] Jørgensen M. E., Borch-Johnsen K., Bjerregaard P. (2008). A cross-sectional study of the association between persistent organic pollutants and glucose intolerance among Greenland Inuit. Diabetologia.

[49] Turyk M, Anderson H, Knobeloch L, Imm P, Persky V (2009). Organochlorine exposure and incidence of diabetes in a cohort of great lakes sport fish consumers. Environ. Health Perspect..

[50] Rignell-Hydbom A (2009). Exposure to p,p’-DDE: A risk factor for type 2 diabetes. PLoS One.

[51] Tyrrell JB, Hafida S, Stemmer P, Adhami A, Leff T (2017). Lead (Pb) exposure promotes diabetes in obese rodents. J. Trace Elem. Med. Biol..

[52] Leff, T., Stemmer, P., Tyrrell, J. & Jog, R. Diabetes and Exposure to Environmental Lead (Pb). *Toxics* **6** (2018). 10.3390/toxics6030054 PMC6161143 30200608

[53] Forte G (2013). Blood Metals Concentration in Type 1 and Type 2 Diabetics. Biol. Trace Elem. Res..

[54] Hectors TLM (2011). Environmental pollutants and type 2 diabetes: a review of mechanisms that can disrupt beta cell function. Diabetologia.

[55] Ekong EB, Jaar BG, Weaver VM (2006). Lead-related nephrotoxicity: a review of the epidemiologic evidence. Kidney Int..

[56] Tsaih S-W (2004). Lead, diabetes, hypertension, and renal function: the normative aging study. Environ. Health Perspect..

