# Supplementary material for: Examining environmental contaminant mixtures among adults with type 2 diabetes in the Cree First Nation communities of Eeyou Istchee, Canada
Source: Sci Rep. 2019 Nov 4;9:15909. doi: 10.1038/s41598-019-52200-x (PMC6828760; doi:10.1038/s41598-019-52200-x)
Supplement: Supplementary file 1 — Supplementary information [file 41598_2019_52200_MOESM1_ESM.pdf]

Supplementary information:

Examining environmental contaminant mixtures among adults with type 2 diabetes in the Cree First Nation communities of *Eeyou Istchee*, Canada

*Aleksandra M Zuk[1],
Leonard J Tsuji[1],
Evert Nieboer[2],
Ian D Martin[1],
Eric N Liberda[3]

Author affiliations:
[1]Department of Physical and Environmental Sciences, University of Toronto Scarborough, Toronto, Ontario, Canada.
[2]Biochemistry and Biomedical Sciences, McMaster University, Hamilton, Ontario, Canada.
[3]School of Occupational and Public Health, Ryerson University, Toronto, Ontario, Canada.

Supplemental Figure S1

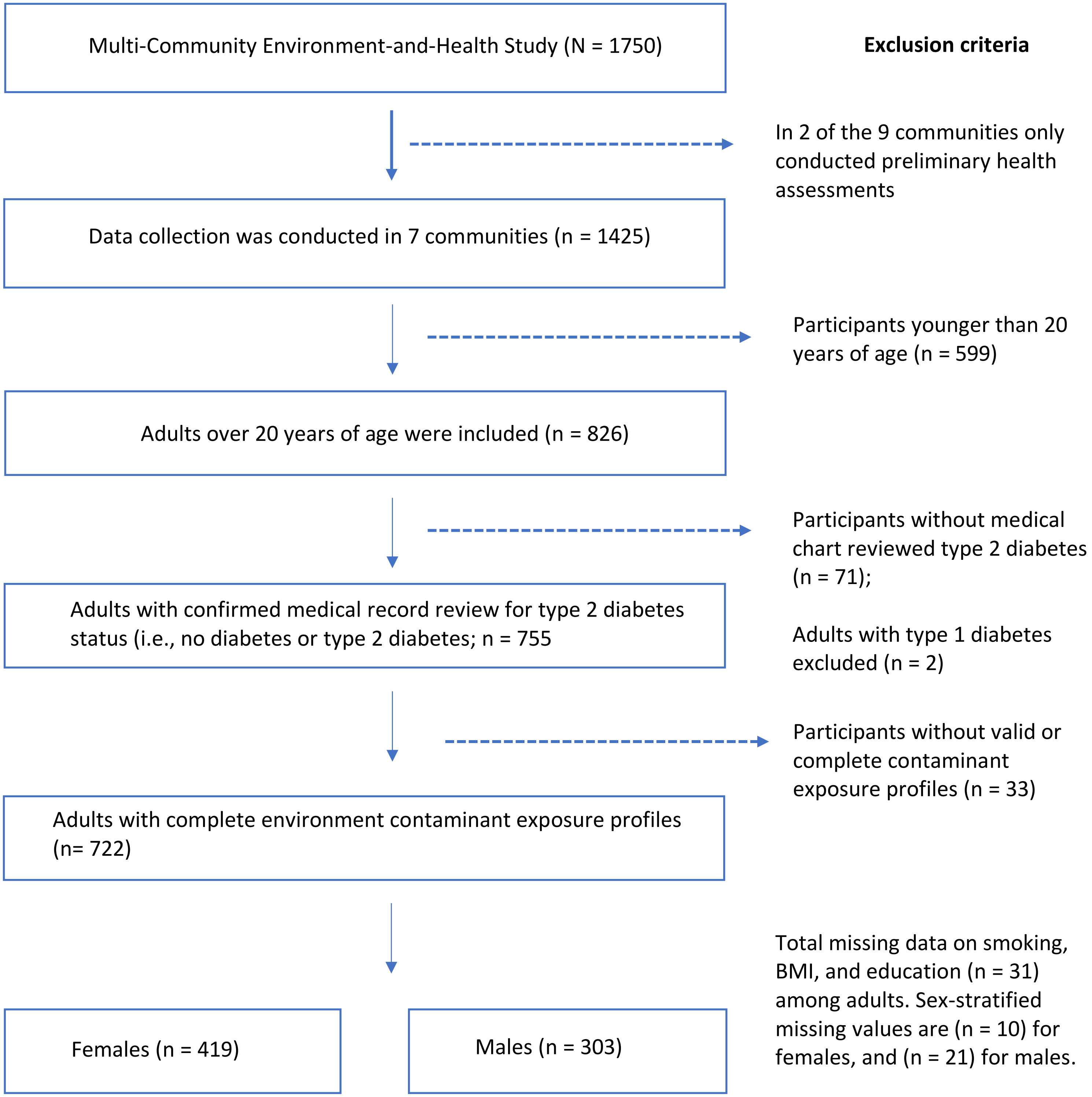

Supplementary Table S4: Multivariable adjusted prevalence ratios (PR; 95% Confidence Intervals) for type 2 diabetes mellitus and single-pollutant models among sex-stratified adults over 20 years of age using data from the *Nituuchischaayihtitaau Aschii* – Multi-Community Environment-and-Health Study, 2005-2009

**Males**	**Prevalence Ratio (PR) †**	**95% CIL**	**95% CIH**	**P value***
**Lead [Pb]**				
Model 1	0.3872	0.1945	0.7705	**0.0069 ***
Model 2	0.4064	0.2040	0.8098	**0.0105 ***
**Dichlorodiphenyltrichloroethane [*p,p'*-DDT]**				
Model 1	1.2461	0.6866	2.2616	0.4693
Model 2	1.5159	0.8136	2.8242	0.1901
**Females**	**Prevalence Ratio (PR)†**	**95% CI**	**95% CI**	**P value***
**Lead [Pb]**				
Model 1	0.7115	0.4640	1.09	0.1187
Model 2	0.6760	0.4402	1.0380	0.0736
**Dichlorodiphenyltrichloroethane [*p,p'*-DDT]**				
Model 1	2.1471	1.4116	3.2659	**0.0004 ***
Model 2	1.9835	1.2812	3.0707	**0.0021 ***

*Significance ($p < 0.05$)

Model 1: adjusted for age;
Model 2: Model 1, plus lipids

† Using SAS PROC GENMOD procedures, adjusted prevalence ratios were estimated using modified Poisson regression with robust error variance. Regressing prevalent T2DM on detectable vs non-detectable Pb and DDT levels.
